# The walnut-derived peptide TW-7 improves mouse parthenogenetic embryo development of vitrified MII oocytes potentially by promoting histone lactylation

**DOI:** 10.1186/s40104-024-01045-0

**Published:** 2024-06-11

**Authors:** Yaozong Wei, Bo Pan, Jianpeng Qin, Beijia Cao, Tianyi Lv, Jiangfeng Ye, Ao Ning, Kunlin Du, Xiangyi Chen, Shuqi Zou, Shengqin Zang, Guozhi Yu, Tianzeng Song, Qiuxia Liang, Guangbin Zhou

**Affiliations:** 1 State Key Laboratory of Swine and Poultry Breeding Industry, Key Laboratory of Livestock and Poultry Multi-Omics, Ministry of Agriculture and Rural Affairs, and Farm Animal Genetic Resources Exploration and Innovation Key Laboratory of Sichuan Province, College of Animal Science and Technology, Sichuan Agricultural University, Chengdu, 611130 China; 2 College of Life Science, Sichuan Agricultural University, Ya’an, 625014 Sichuan China; 3 Institute of Animal Science, Xizang Academy of Agricultural and Animal Husbandry Science, Lhasa, 850009 Xizang China

**Keywords:** Histone lactylation, Oocyte, TW-7, Vitrification, Zygotic genome activation

## Abstract

**Background:**

Previous studies have shown that the vitrification of metaphase II (MII) oocytes significantly represses their developmental potential. Abnormally increased oxidative stress is the probable factor; however, the underlying mechanism remains unclear. The walnut-derived peptide TW-7 was initially isolated and purified from walnut protein hydrolysate. Accumulating evidences implied that TW-7 was a powerful antioxidant, while its prospective application in oocyte cryopreservation has not been reported.

**Result:**

Here, we found that parthenogenetic activation (PA) zygotes derived from vitrified MII oocytes showed elevated ROS level and delayed progression of pronucleus formation. Addition of 25 μmol/L TW-7 in warming, recovery, PA, and embryo culture medium could alleviate oxidative stress in PA zygotes from vitrified mouse MII oocytes, furtherly increase proteins related to histone lactylation such as LDHA, LDHB, and EP300 and finally improve histone lactylation in PA zygotes. The elevated histone lactylation facilitated the expression of minor zygotic genome activation (ZGA) genes and preimplantation embryo development.

**Conclusions:**

Our findings revealed the mechanism of oxidative stress inducing repressed development of PA embryos from vitrified mouse MII oocytes and found a potent and easy-obtained short peptide that could significantly rescue the decreased developmental potential of vitrified oocytes, which would potentially contribute to reproductive medicine, animal protection, and breeding.

**Supplementary Information:**

The online version contains supplementary material available at 10.1186/s40104-024-01045-0.

## Background

Oocyte cryopreservation has been widely used in reproductive medicine [1, 2] and animal breeding [3]. However, the blastocyst formation rate of vitrified MII oocytes after PA or in vitro fertilization (IVF) is markedly lower than the fresh oocytes, 5.60%–16.70% in humans [4, 5], 7.67%–11.60% in pigs [6–8], 7.80%–10.10% in cattle [9, 10], 4.60%–9.20% in sheep [11] and 25.46%–63.76% in mice [12–15], which significantly hinders its commercial application. Therefore, it’s urgent to improve the developmental potential of vitrified oocytes. Accumulating researches have shown that oxidative stress was one important factor restricting vitrified oocytes development [16, 17]. Under normal physiological conditions, reactive oxygen species (ROS) are in equilibrium with the intracellular antioxidant defense system. When subjected to external stimuli (e.g., low temperature), the intracellular redox homeostasis is destroyed, and the excess ROS generation, which can attack DNA and protein (e.g., various enzymes), resulting in DNA mutation and protein inactivation [18, 19]. Supplementation with exogenous antioxidants has been recognized as one of the important strategies to improve vitrified oocyte quality and developmental potential [20, 21]. However, how ROS affects the development of embryo derived from vitrified oocyte need to be revealed, and more cost-effective, and easily obtainable antioxidants also need to be explored.

ZGA is critical for preimplantation embryo development. During the initial stage of zygote formation, the genome remains transcriptionally silent [22]. The proteins and RNAs required for development are completely provided by the maternal gamete [23], and then ZGA is initiated during the maternal-to-zygotic transition (MZT). As the first post-fertilization transcriptional event, ZGA is usually divided into two stages: minor ZGA and major ZGA [24]. Minor ZGA occurs at the S stage of the zygote and the G_1_ stage of the early 2-cell embryo, and major ZGA occurs at the middle to late stage of the 2-cell embryo [25]. Inhibition of the minor ZGA by drugs leads to abnormal activation of the mouse zygotic genome and ultimately to embryo developmental arrest [26]. Therefore, whether the developmental arrest induced by vitrification is due to abnormal minor ZGA initiation needs to be further explored.

Recently, Zhang et al. [27] have identified lactate-derived histone lysine lactylation, as a novel epigenetic modification, that can activate the transcription of target genes. To date, researchers have found that histone lactylation modifications played a critical role in tumorigenesis [28], somatic cell reprogramming [29], inflammation [30], neurological diseases [31], and embryo development [32, 33]. Studies showed that histone lactylation could impact cell cycle transition in cancer cells [34] and ZGA gene expression in both embryonic stem cells and mammalian preimplantation embryos [35, 36]. Meanwhile, at the early cleavage stage, the energy supply of embryos greatly depends on pyruvate and lactate metabolism [37, 38], whereas vitrification brought about disturbed energy metabolism in mouse MII oocytes [16, 39]. In addition, studies have shown that dramatically increased ROS can repress the production of glycolytic products such as lactate [40, 41]. Accordingly, it remains to be addressed whether the elevated ROS in vitrified MII oocytes restrains their development potential by affecting the level of histone lactylation modifications.

The walnut-derived peptide TW-7 (Thr-Trp-Leu-Pro-Leu-Pro-Arg) is a short peptide, initially isolated and purified from walnut protein hydrolysate. Accumulating evidences implied that TW-7 was a powerful antioxidant [42–44]. For example, in PC-12 cells, TW-7 can inhibit ROS production and protect mitochondrial function by regulating antioxidant enzymes, such as glutathione peroxidase (GPx) [44]. Given that TW-7 can be easily synthesized at relatively low cost, it’s of great value to investigate the TW-7 supplementation for improving embryo development derived from vitrified MII oocytes.

## Materials and methods

The walnut-derived peptide TW-7 was synthesized by Jiangsu Ji Tai Peptide Industry Science and Technology Co. Ltd. (Yancheng, China) with a purity of 99.8%. Unless otherwise indicated, all chemicals were purchased from Sigma-Aldrich (St. Louis, MO, USA). All experimental procedures were conducted in strict accordance with the regulations of the Animal Ethics and Welfare Committee (AEWC) of Sichuan Agricultural University, China (Approval code: AEWC2016, January 6, 2016).

### Oocyte collection

Female ICR mice were purchased from Dashuo Company, Chengdu, China. Female mice (6–8 weeks old) were prepared after 2 weeks of acclimatization. The duration of light was controlled at 14 h, the ambient temperature was 18–25 °C, and the humidity was 50%–70%. Procedure of superovulation: each female mouse was injected with 10 IU equine chorionic gonadotropin (PMSG, Ningbo Second Hormone Factory, Ningbo, China) and 10 IU human chorionic gonadotropin (hCG, Ningbo Second Hormone Factory, Ningbo, China) 48 h later. 12–14 h after hCG injection, female mice were euthanized by cervical dislocation. Cumulus oocyte complexes (COCs) were collected from the oviducts. Cumulus cells were dispersed in 300 IU/mL of hyaluronidase (3–5 min) and washed three times in M2 medium to reject the oocytes with substandard quality.

### Oocyte vitrification and warming

Oocyte vitrification was performed by the open-pulled straw (OPS) method, and OPS was made as described previously [12]. Briefly, the straws (0.25 mL, IMV, France) were heat-softened and quickly pulled to get a length of 3 cm, with an inner diameter of about 0.10 mm and an outer diameter of about 0.15 mm. Vitrification-warming procedures were performed as described previously [45]. For vitrification, 8–10 oocytes were aspirated with OPS straws and exposed to phosphate buffer saline (PBS) containing 10% ethylene glycol and 10% dimethyl sulfoxide for 30 s. Then, the oocytes were transferred to PBS containing 15% ethylene glycol, 15% dimethyl sulfoxide, 300 g/L Ficoll, 0.5 mol/L sucrose, and 3 g/L fetal bovine albumin for 25 s. Finally, the straws containing oocytes were plunged into liquid nitrogen quickly. In terms of oocyte warming, oocytes were rinsed in a warming medium (M2 medium containing 0.5 mol/L sucrose) for 5 min, and then, washed three times in the M2 medium.

### PA of oocyte and in vitro culture (IVC)

PA of oocyte and IVC were performed following previously validated protocol [46]. Briefly, oocytes were resuscitated in M2 medium (recovery medium) for 1 h before parthenogenetic activation, and then incubated to parthenogenetic activation A medium (calcium-free HTF solution containing 10 mmol/L SrCl_2_ and 2 μg/mL cytochalasin D) for 2.5 h, and followed by a 3.5 h incubation in parthenogenetic activation B medium (HTF solution containing 2 μg/mL cytochalasin D). Finally, oocytes were cultured in KSOM-AA. The formation rates of 2-cell embryos, 4-cell embryos, and blastocysts were measured at 24, 48, and 96 h post activation (hpa), respectively.

### TW-7, H_2_O_2_, oxamate, and lactate treatment

TW-7 (25, 50, and 100 μmol/L), oxamate (5 mmol/L, a glycolysis inhibitor that reduces intracellular lactate levels [47, 48], HY-W0130032A, MedChemExpress, Monmouth Junction, NJ, USA), and lactate (5 mmol/L, a histone lactation agonist [28], HY-B2227B, MedChemExpress) were supplemented in the warming, recovery, PA and IVC medium. Hydrogen peroxide (H_2_O_2_, 100 μmol/L) was added to the IVC medium as an oxidative stress inducer for 1 h [49].

### ROS level detection

ROS measurement was conducted according to the procedure described previously [50]. PA zygotes (9 hpa) were incubated in an M2 medium containing 20 μmol/L DCFH-DA (C2938, Invitrogen, Carlsbad, CA, USA) for 30 min (37 °C, 100% humidity, and 5% CO_2_ concentration), and then washed three times in M2 medium containing 3 mg/mL bovine serum albumin. Finally, the embryos were mounted in 20 μL M2 drop on a slide. Fluorescence images were captured with a fluorescence microscope (BX53, Olympus, Tokyo, Japan). The fluorescence intensity (optical density) was analyzed using Image J software (version 1.48, Bethesda, MD, USA) and used as a ROS level assessment.

### 5-Ethynyl-20-deoxyuridine (EdU) staining

The ratio of zygotes at the S-phase was analyzed according to the instructions of the EdU Assay Kit (C10310-3, Ruibo Biotechnology Co., Ltd., Guangzhou, China). Treated zygotes (9 hpa) were placed on slides containing 10 μL DAPI (Vector Laboratories Inc., Burlingame, CA, USA) and covered with a glass cover. Finally, Fluorescence images were captured with a fluorescence microscope (BX53, Olympus, Tokyo, Japan), with green fluorescence indicating EdU labeling.

### 5-Ethynyl uridine (EU) staining

The detection of the zygotic new transcripts followed the manufacturer's instructions for the EU assay kit (C10316-1, Ruibo Biotechnology Co., Ltd, Guangzhou, China). Treated zygotes (7–11 hpa) were placed on slides containing 10 μL DAPI (Vector Laboratories Inc., Burlingame, CA, USA) and covered with a glass cover. Finally, Fluorescence images were captured using a fluorescence microscope (BX53, Olympus, Tokyo, Japan), with red fluorescence indicating EU labeling.

### Immunofluorescence staining

Immunofluorescence staining was performed according to the following procedures. PA zygotes (9 hpa) were fixed with 4% (w/v) paraformaldehyde for 20 min at room temperature and then permeabilized by PBS containing 1% TritonX-100 for 20 min at room temperature. After that, zygotes were antigen-blocked with PBS containing 1% BSA for 30 min at room temperature and then transferred to the primary antibody and incubated at 4 °C overnight. The next day, zygotes were transferred to the secondary antibody and incubated at 37 °C for 1 h. The zygotes were washed three times for 10 min each by PBS containing 0.01% Triton X-100 and 0.1% Tween 20 after both primary and secondary antibody incubation. Finally, the zygotes were placed on slides containing 10 μL DAPI (Vector Laboratories Inc., Burlingame, CA, USA) and covered with a glass cover. Fluorescence images were captured with a fluorescence microscope (BX53, Olympus, Tokyo, Japan). The fluorescence intensity (optical density) was analyzed by Image J software (version 1.48, Bethesda, MD, USA) and used as a protein expression level assessment.

The primary antibodies used for immunofluorescence were LDHA (rabbit, 19987-1-AP, Proteintech, 1:100), LDHB (rabbit, 14824-1-AP, Proteintech, 1:200), EP300 (rabbit, bs-6954R, Bioss, 1:200), HDAC3 (rabbit, 10255-1-AP, Proteintech, 1:200), Pan-Kla (rabbit, PTM-1401, PTM BIO, 1:200), H3K9la (rabbit, PTM 1419RM, PTM BIO, 1:200), H3K18la (rabbit, PTM 1419RM, PTM BIO, 1:200), H4K12la (rabbit, PTM-1411RM, PTM BIO, 1:200). Secondary antibodies were CoraLite488-conjugated goat anti-rabbit IgG (SA00013-2, Proteintech, 1:400), CoraLite594-conjugated goat anti-rabbit IgG (BF03008, Proteintech, 1:400).

### Quantitative reverse transcription PCR (qRT-PCR)

The qRT-PCR was conducted as previously reported [51]. Briefly, in each group, the TransScript Uni Cell to cDNA Synthesis SuperMix for qPCR kit (AC301, TransGen Biotech, China) was used to obtain the total complementary DNA (cDNA) from PA zygotes (9 hpa). Next, cDNA was quantified by qRT-PCR under standard conditions on a CFX Connect Real-Time Detection System (Bio-Rad, Hercules, CA, USA) using the TransStart Tip Green qPCR SuperMix kit (AQ601, TransGen Biotech, China). The cycle threshold (Ct) used to calculate the relative expression was the average of three replicates and was normalized to the expression of the reference gene (*Gapdh*). The relative mRNA expression level was calculated using the 2^−ΔΔCt^ method [52]. The primer sequences used are shown in Table 1.

**Table 1:** Primers used for qRT–PCR analysis

Gene	Forward (5′→3′)	Reverse (5′→3′)	Accession ID
*Gapdh*	CATGGCCTTCCGTGTTCCTA	GCCTGCTTACCACCTTCTT	NM_008084.3
*Sod1*	CACTCTAAGAAACATGGTGG	GATCACACGATCTTCAATGG	NM_011434.2
*Sod2*	AAGGGAGATGTTACAACTCAGG	GCTCAGGTTTGTCCAGAAAATG	NM_011434.2
*Nrf2*	GTCTTCACTGCCCCTCATC	TCGGGAATGGAAAATAGCTCC	NM_010902.4
*Hmga1*	GGTCGGGAGTCAGAAAGAGC	ATTCTTGCTTCCCTTTGGTCG	NP_057869
*Prr5*	TGGAACAGTATCCATAACGGAGT	CGCCCTCGTTGAGGATGAA	NM_026538
*Bcl2-1*	GACAAGGAGATGCAGGTATTGG	TCCCGTAGAGATCCACAAAAGT	NM_009743

### Western blot

One hundred PA zygotes (9 hpa) were cleaved in RIPA lysate (AR0102, BOSTER, China) containing phosphatase inhibitors (AR1183, BOSTER, China) and protease inhibitors (AR1182-1, BOSTER, China) for 10 min on ice. 5 × SDS-PAGE were added and boiled for 10 min to denature the protein. Gels were prepared using the PAGE Gel Fast Preparation Kit (PG113, Epizyme, China). Electrophoresis was performed after the addition of the samples, and the proteins were transferred to a PVDF membrane (Roche, Basel, Switzerland) and subsequently blocked in 5% skimmed milk diluted in TBST for 2 h. The membrane was then incubated overnight at 4 °C exposed to rabbit anti-LDHB antibody (14824-1-AP, Proteintech, 1:20,000) and rabbit anti-β-tubulin antibody (PTG-10068-1-AP, Proteintech, 1:1,000). The membrane was washed three times for 10 min in TBST and then incubated with the HRP conjugate secondary antibody (S0001, Affinity, 1:3,000) at room temperature for 1 h. Then the membrane was washed three times using TBST and finally performed with ECL and Western blot analyzer system. Gray values were quantified by Image J software.

### RNA sequencing and analysis

Sequencing procedure: 20 PA zygotes (9 hpa) from each group were collected and stored at −80 °C in PCR tubes containing 3 µL of single-cell Smart-Seq lysate. Subsequently, the nucleic acid sequence with Oligo dT was reverse transcribed to form 1^st^ cDNA, followed by PCR-cDNA amplification, purification of the amplified product, and library construction. The library construction included fragmentation, end repair, the addition of "A" splices, and library quality control. The Illumina platform was used for sequencing, with the sequencing strategy PE150.

Analysis procedure: the raw sequencing data (Raw Reads) obtained from Hiseq sequencing was processed to obtain high-quality sequences (Clean Reads) by removing low-quality sequences and adapter contamination. All subsequent analyses were based on Clean Reads. The analysis process was divided into three parts: quality control of the sequencing data, data alignment analysis, and in-depth analysis of the transcriptome. Sequencing data quality control included filtering the sequences obtained from sequencing, evaluating the quality of sequencing data, and calculating the length distribution of sequences, etc. Data alignment analysis referred to aligning the sequences to the genome. Classifying and analyzing of the features was based on different genome annotation information, and calculating the corresponding expression levels. Transcriptome in-depth analysis included differentially expressed analysis, alternative splicing analysis, and personalized analysis such as the prediction of new transcripts.

Differentially expressed genes (DEGs), ZGA genes, and maternal genes analysis: DEGs with a *P* < 0.05 and fold change > 1.5 were identified using DESeq2. The GO terms of DEGs were analyzed using Metascape [53]. ZGA genes and maternal genes were derived from further analysis based on DEGs. As described by the previous researchers [54], ZGA genes were defined as genes that were not expressed or expressed at a low level (FPKM < 5) during the MII stage of oocytes but were up-regulated (FPKM > 5, fold change > 3) in zygotes. Maternal genes were defined as genes that were highly expressed during MII oocytes (FPKM > 5) but down-regulated (fold change > 3) in the zygotes [54].

### Statistical analysis

Statistical analyses were performed by one-way ANOVA using post hoc Fisher's least significant difference (LSD) test of SPSS statistical software (v.20.0, IBM, Chicago, IL, USA). Graphs were plotted by GraphPad Prism 9 software (GraphPad, La Jolla, CA, USA). The data were presented in the form of mean ± standard error (mean ± SEM). All experiments were replicated at least three times. Only *P* < 0.05 was considered statistically significant. Prior to ANOVA, several percentage data, including developmental rate, the formation of pronucleus, the positive proportion of EU staining, and the percentage in S phase, were arcsine transformed.

## Results

### TW-7 improved the developmental potential of the PA embryos from vitrified mouse MII oocytes

**Fig. 1:**
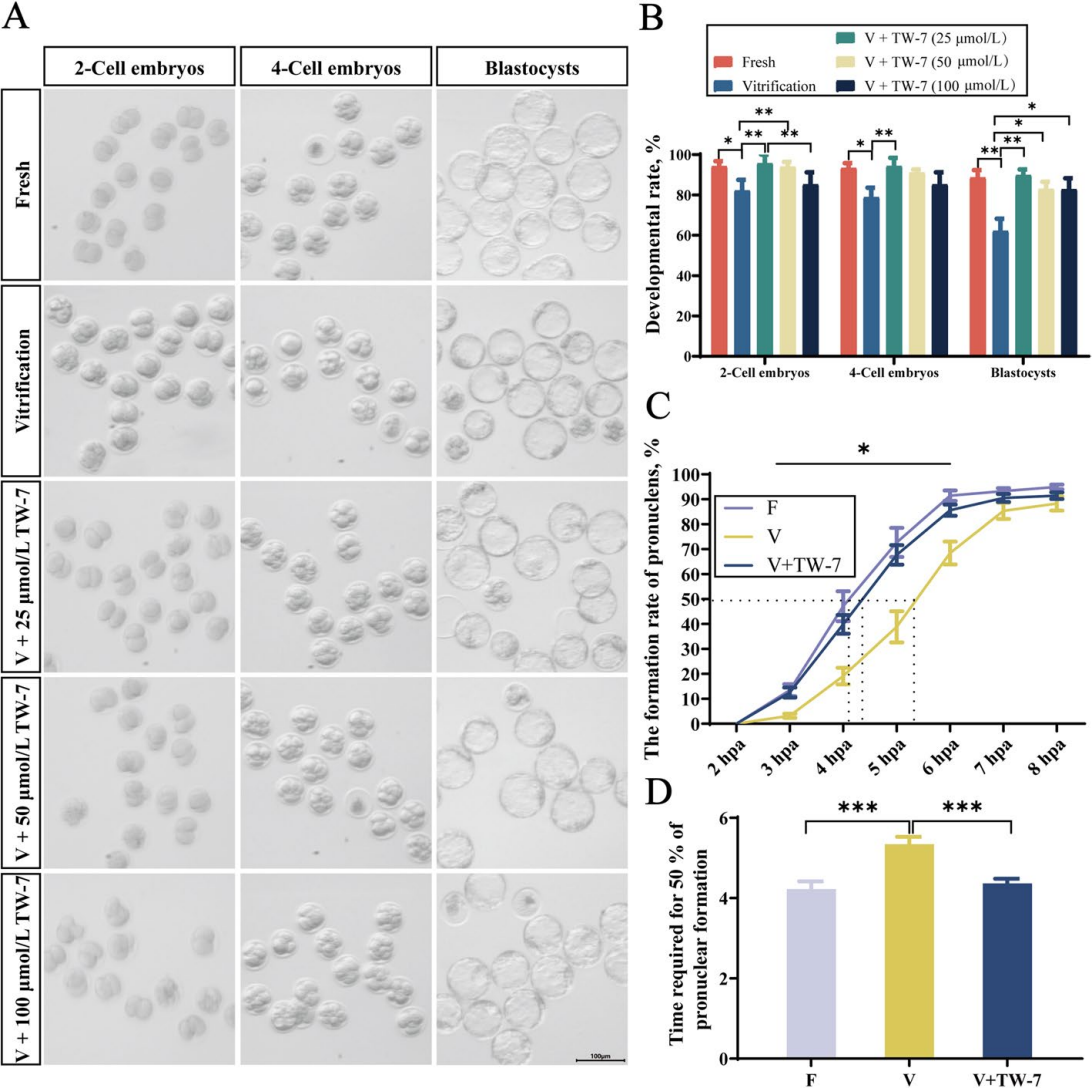
TW-7 improves the developmental potential of the PA embryos from vitrified mouse MII oocytes. **A** Representative images of 2-cell embryos, 4-cell embryos, and blastocysts in the Fresh, Vitrification, V + 25 μmol/L TW-7, V + 50 μmol/L TW-7 and V + 100 μmol/L TW-7 groups. Scale bar, 100 μm. **B** The percentage of embryo development rate of oocytes in Fresh, Vitrification, V + 25 μmol/L TW-7, V + 50 μmol/L TW-7, and V + 100 μmol/L TW-7 groups. Fresh (*n* = 116), Vitrification (*n* = 95), V + 25 μmol/L TW-7 (*n* = 80), V + 50 μmol/L TW-7 (*n* = 84), V + 100 μmol/L TW-7 (*n* = 85). **C** The rate of pronuclear formations in the Fresh, Vitrification, and V + TW-7 groups from 2 to 8 hpa. The dotted lines indicate the time after activation corresponding to the 10% and 50% rates of pronuclear formation in each group. **D** Time required for 50% of pronuclear formation. Fresh (*n* = 146), Vitrification (*n* = 143), V + TW-7 (*n* = 127). Data in (**B**), (**C**), and (**D**) were presented as mean percentage (mean ± SEM) of at least three independent experiments. ^*^P < 0.05, ^**^P < 0.01, ^***^P < 0.001

To determine whether TW-7 could ameliorate the developmental potential of vitrified mouse mature oocytes, we supplemented warming, recovery, PA, and embryo culture medium with TW-7 in different concentrations (25, 50, and 100 μmol/L), then evaluated subsequent developmental competence after parthenogenetic activation. As shown in Fig. 1A and B, compared with the fresh group, the developmental rates of 2-cell embryos, 4-cell embryos, and blastocysts were all significantly lower in the vitrification group. When the above-mentioned culture medium was supplemented with 25 μmol/L TW-7, there was a significant increase in the embryo development rates at all three stages (*P* < 0.05), and were comparable to those of fresh groups (*P* > 0.05). When the treatment concentration was increased to 50 and 100 μmol/L, the blastocyst rates also recovered to the equal level of the fresh group (*P* > 0.05). However, there was no obvious change in the rate of 2-cell embryos between the 100 μmol/L treatment group and vitrification group (*P* > 0.05), while no significant difference was observed between 50 and 100 μmol/L treatment groups and vitrification groups. The above results indicated that the addition of 25 μmol/L TW-7 could rescue the suppressed preimplantation embryo development, but TW-7 and embryo development potential did not exhibit a dose-dependent relationship from 25 to 100 μmol/L. Therefore, TW-7 at 25 μmol/L was selected for further study.

Our previous study [12] suggested that oocyte vitrification could lead to a delay in the progression of pronucleus formation, which was an important factor causing embryo development suppression. To explore whether TW-7 could rescue pronucleus formation retardation, we assessed the pronucleus formation rates during the period of 2–8 hpa by using the stereomicroscope. As shown in Fig. 1C, at 2 hpa, no pronuclei were observed in zygotes of all groups. The pronuclei began to form from 3 hpa in all groups, however, the pronucleus formation rates in the vitrification group were significantly lower than those of the fresh group from 3 to 6 hpa (*P* < 0.05). When the vitrification group was supplemented with TW-7, the pronucleus formation rates were significantly increased (*P* < 0.05) and were comparable to those of the fresh group (*P* > 0.05). From 7 to 8 hpa, the rates of pronucleus formation showed no difference in all three groups (*P* > 0.05). As shown in Fig. 1D, the time required for 50% pronucleus formation of PA zygotes in the vitrification group was significantly longer than that of the fresh group and V + TW-7 group (*P* < 0.05). There was no statistical difference in the latter two groups (*P* > 0.05). The above results suggested that the supplement of TW-7 could promote pronucleus formation of PA zygotes derived from vitrified mouse MII oocytes and subsequent embryo development.

### TW-7 addition promoted the G_1_/S transition and ZGA initiation of the PA zygotes derived from vitrified mouse MII oocytes

To further explore the mechanism by which TW-7 enhances the developmental potential of vitrified mouse MII oocytes, we firstly examined the G_1_/S transition of zygotes. A previous study [12] showed that mouse zygotes exited the G_1_ phase into the S phase at 6 hpa, and the proportion of the S phase zygotes reached the highest value at 9 hpa, then gradually exited the S phase into the G_2_ phase thereafter. Therefore, we examined the proportion of PA zygotes in the S phase at 9 hpa. EdU staining (Fig. S1A) showed that the percentage of zygotes in the S phase was significantly lower in the vitrification group than in the fresh group (*P* < 0.05), which returned to the relative level with the fresh group after the addition of TW-7 (*P* > 0.05). These results demonstrated that TW-7 could promote the G_1_/S phase transition of the PA zygotes derived from vitrified mouse MII oocytes.

**Fig. 2:**
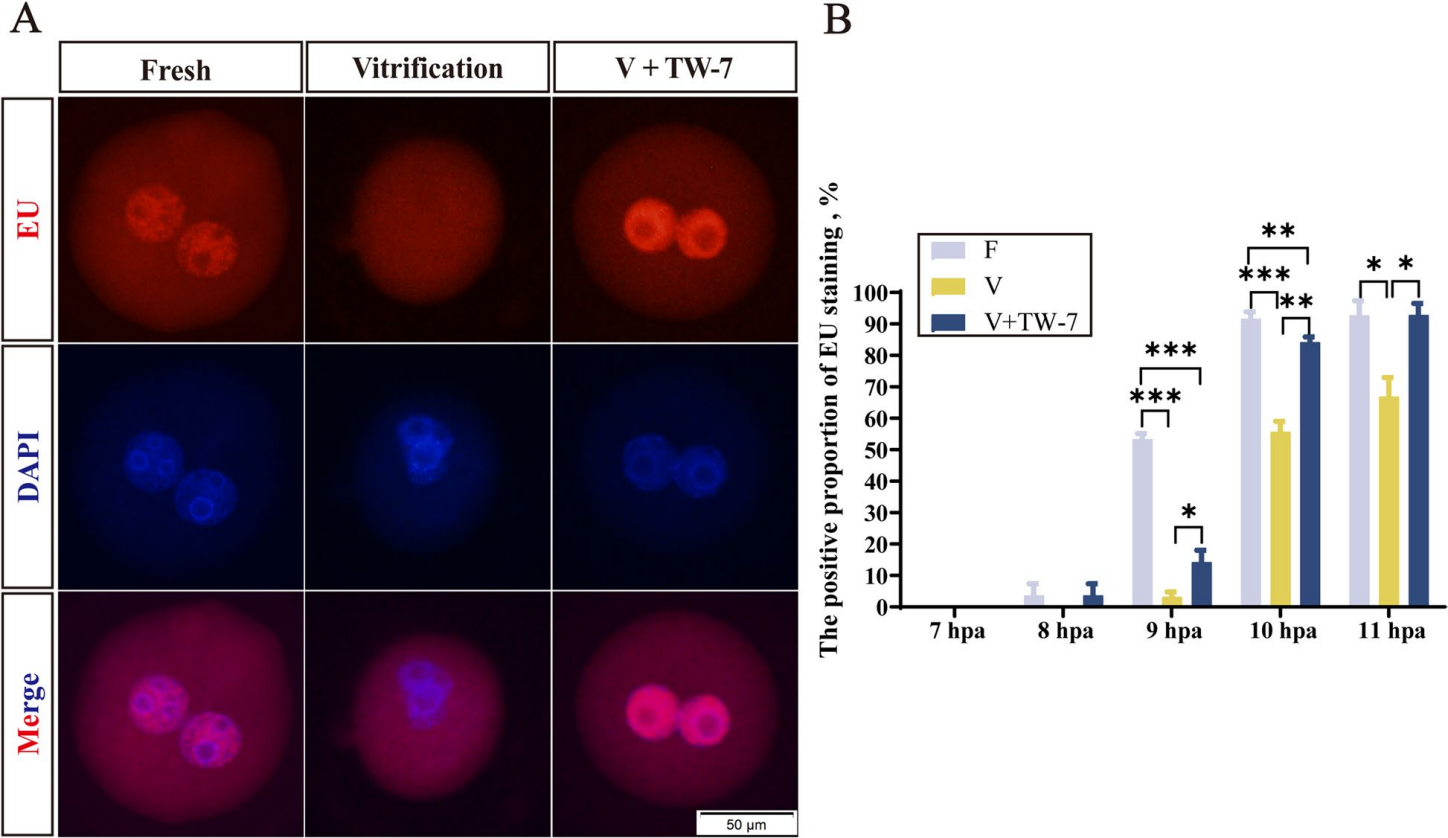
TW-7 promotes the new transcripts generation of the PA zygotes derived from vitrified mouse MII oocytes. **A** Representative images of EU staining in PA zygotes of Fresh, Vitrification, and V + TW-7 groups. Scale bar, 50 μm. **B** The positive proportion of EU staining in PA zygotes of Fresh, Vitrification, and V + TW-7 groups. Fresh (*n* = 225), Vitrification (*n* = 220), V + TW-7 (*n* = 192). Data in (**B**) were presented as mean percentage (mean ± SEM) of at least three independent experiments. ^*^P < 0.05, ^**^P < 0.01, ^***^P < 0.001

ZGA is critical for preimplantation embryo development, and minor ZGA initiates in the S stage of the zygotes. EU staining was conducted to detect the minor ZGA of PA zygotes. The results (Fig. 2A and B) showed that no EU fluorescence was observed in zygotes at 7 hpa. At 8 hpa, the EU staining signal appeared in the fresh and V + TW-7 groups, and there was no distinct difference between them (*P* > 0.05). At 9 hpa, the zygotes of the vitrification group started to show positive EU staining signal, but the rate of positive staining of zygotes was dramatically lower than the fresh and V + TW-7 groups (*P* < 0.05), which continued to 11 hpa. By contrast, the rate of the V + TW-7 group was notably lower than the fresh group at 9–10 hpa (*P* < 0.05), and finally rose to a comparable level to the fresh group at 11 hpa (*P* > 0.05). These data indicated that vitrification suppressed the generation of new transcripts in zygotes, which could be improved by the addition of TW-7.

**Fig. 3:**
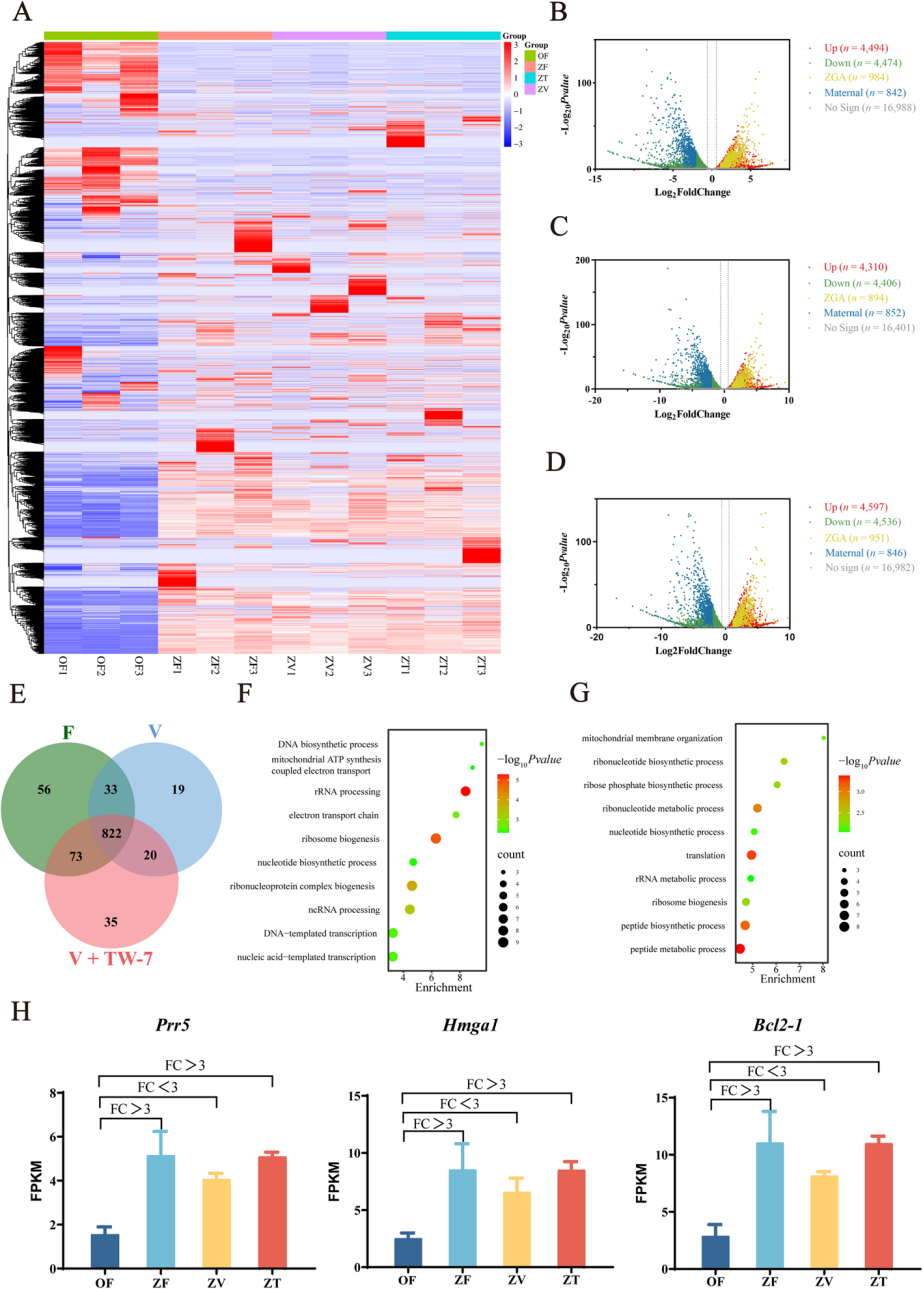
TW-7 promotes minor ZGA initiation in PA zygotes from vitrified mouse MII oocytes. **A** Heatmap of genes in the MII oocytes and PA zygotes (9 hpa). OF: MII oocytes (0 hpa), ZF: PA zygotes (9 hpa) of fresh group, ZV: PA zygotes (9 hpa) of vitrification group, ZT: PA zygotes (9 hpa) of V + TW-7 group. **B–D** Volcano map of differentially expressed genes in different groups during the development of MII oocytes to the zygote (9 hpa). **E** Venn diagram of activated ZGA genes in zygotes of Fresh, Vitrification, and V + TW-7 groups. **F** GO BP enrichment analysis of 129 ZGA genes suppressed by vitrification at the zygotes. **G** GO BP enrichment analysis of 108 ZGA genes activated by TW-7 at the zygotes. GO, Gene Ontology. BP, Biological Process. **H** Histogram of *Prr5*, *Bcl2-1,* and *Hmga1* expression in MII oocytes and at the zygotic stage

Next, we carried out single-cell RNA sequencing to figure out which ZGA gene expression was affected by vitrification. Considering Gao et al. found that cryopreservation did not affect the transcriptome of MII oocyte [55], we detected the MII oocyte transcriptome of the fresh group and used the data as a control to analyze changes in gene expression levels from MII oocyte to zygotes in fresh, vitrification and V + TW-7 groups. The heatmap of genes (Fig. 3A) indicated that samples of the same group were reproducible, and there were great differences between the transcription levels of MII oocytes and PA zygotes. Further analysis found that the expression of maternal genes between the above-mentioned three groups did not change significantly (Fig. 3B–D). However, there was a great change in the ZGA gene expression, and the upregulated genes in fresh, vitrification and V + TW-7 groups were 984, 894 and 951, respectively (Fig. 3B–D). Compared to the fresh group, vitrification suppressed the activation of 129 ZGA genes (Fig. 3E), which were mainly enriched in RNA processing, ribosome biogenesis, transcription and mitochondrial function (Fig. 3F). The activation of 108 ZGA genes was restored by the addition of TW-7, which were mainly enriched in translation, biosynthesis process, and mitochondrial membrane organization (Fig. 3G). For example, the expression of genes *Prr5*, *Hmga1,* and *Bcl2-1* which were involved in the transcriptional regulation or cellular proliferation were increased in fresh and V + TW-7 groups, but not in the vitrification group (Fig. 3H).

In conclusion, the above results demonstrated that MII oocyte vitrification had a negative impact on two key zygote development events, G_1_/S transition, and ZGA initiation, which could be alleviated by TW-7.

PA zygotes derived from vitrified mouse MII oocytes showed significantly suppressed levels of histone lactylation, which could be rescued by TW-7 addition.

**Fig. 4:**
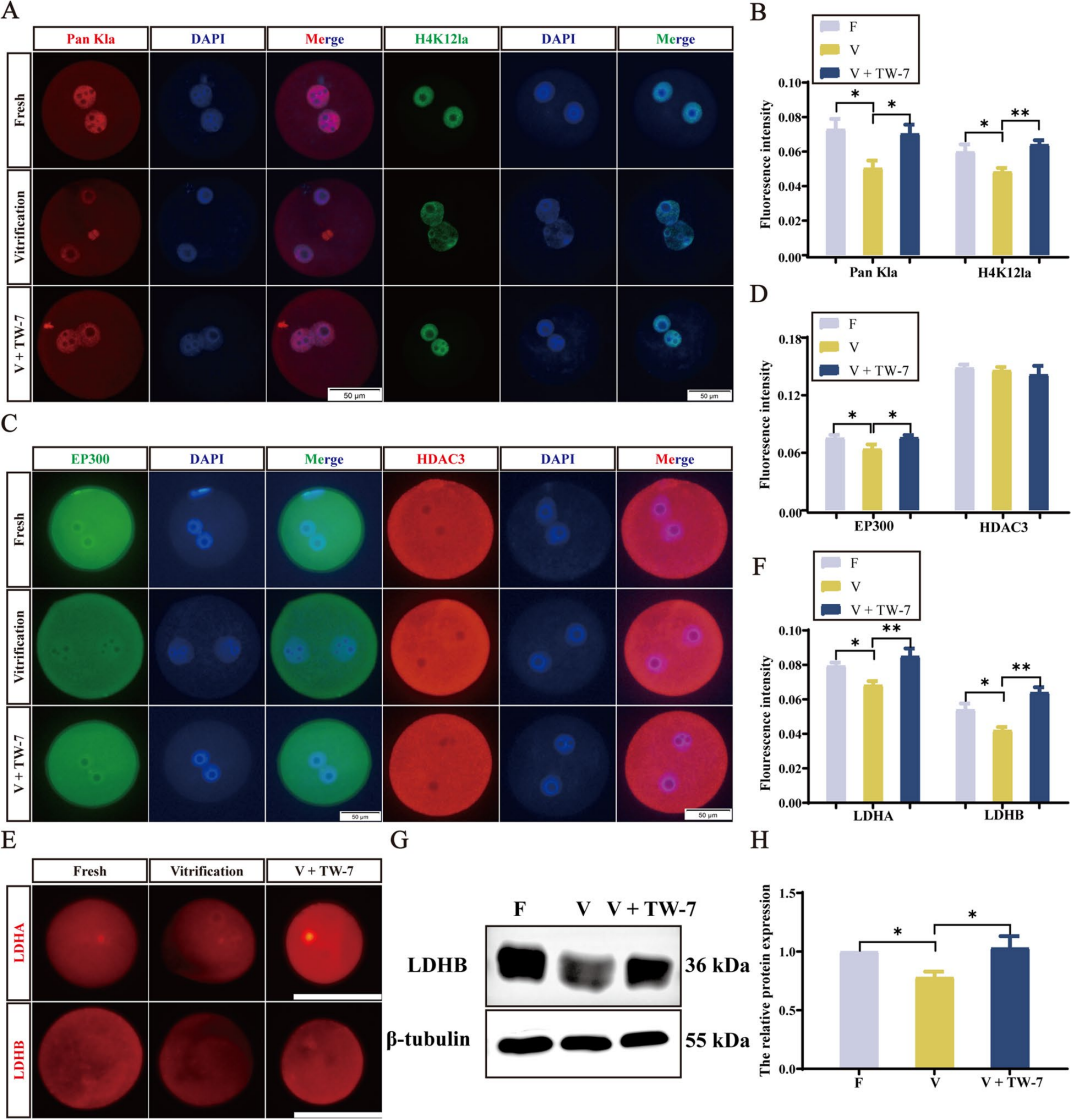
TW-7 regulates the protein expression levels of histone lactylation in PA zygotes derived from vitrified mouse MII oocytes. **A** Representative images of Pan Kla and H4K12la protein expression in PA zygotes of Fresh, Vitrification, and V + TW-7 groups. Scale bar, 50 μm. **B** The fluorescence intensity of Pan Kla and H4K12la in PA zygotes of Fresh, Vitrification, and V + TW-7 groups. Number of PA zygotes involved in Pan Kla immunofluorescence staining: Fresh (*n* = 22), Vitrification (*n* = 24), V + TW-7 (*n* = 27). Number of PA zygotes involved in H4K12la immunofluorescence staining: Fresh (*n* = 50), Vitrification (*n* = 38), V + TW-7 (*n* = 42). **C** Representative images of EP300 and HDAC3 protein expression in PA zygotes of Fresh, Vitrification, and V + TW-7 groups. Scale bar, 50 μm. **D** The fluorescence intensity of EP300 and HDAC3 in PA zygotes of Fresh, Vitrification, and V + TW-7 groups. Number of PA zygotes involved in EP300 immunofluorescence staining: Fresh (*n* = 46), Vitrification (*n* = 49), V + TW-7 (*n* = 44). Number of PA zygotes involved in HDAC3 immunofluorescence staining: Fresh (*n* = 25), Vitrification (*n* = 21), V + TW-7 (*n* = 24). **E** Representative images of LDHA and LDHB protein expression in PA zygotes of Fresh, Vitrification, and V + TW-7 groups. Scale bar, 100 μm. **F** The fluorescence intensity of LDHA and LDHB in PA zygotes of Fresh, Vitrification, and V + TW-7 groups. Number of PA zygotes involved in LDHA immunofluorescence staining: Fresh (*n* = 37), Vitrification (*n* = 38), V + TW-7 (*n* = 38). Number of PA zygotes involved in LDHB immunofluorescence staining: Fresh (*n* = 36), Vitrification (*n* = 31), V + TW-7 (*n* = 37). **G** Representative images of LDHB Western blotting in parthenogenetic zygotes of Fresh, Vitrification, and V + TW-7 groups. **H** The relative grayscale values of LDHB in PA zygotes of the Fresh, Vitrification, V + TW-7 groups. Fresh (*n* = 400), Vitrification (*n* = 400), V + TW-7 (*n* = 400). Data in (**B**), (**D**), (**F**), and (**H**) were presented as mean percentage (mean ± SEM) of at least three independent experiments. ^*^P < 0.05, ^**^P < 0.01

To investigate whether histone lactylation participated in the minor ZGA initiation inhibition of PA zygotes from vitrified MII oocytes, we firstly examined the histone lysine lactylation levels in PA zygotes (9 hpa) in different treatment groups. Detection of the general level of histone lactylation using an anti-Pan-Kla found that the immunofluorescence intensity of Pan Kla of the vitrification group was significantly lower than that of the fresh group (*P* < 0.05) (Fig. 4A and B). After the addition of TW-7 in the vitrification group, the Pan Kla fluorescence intensity increased significantly (*P* < 0.05) and was not significantly different (*P* > 0.05) from the fresh group. Immunofluorescence detected the lactylation levels of three histone lysine sites, H3K9la, H3K18la, and H4K12la, and found that the trend of H4K12la levels was consistent with Pan Kla in three groups (Fig. 4A and B). The H3K18la levels showed no difference in the three groups (*P* > 0.05), and the immunofluorescence intensity of H3K9la was significantly higher (*P* < 0.05) in the V + TW-7 group than the other two groups (Fig. S2A and B). These results implied that TW-7 could not only rescue the suppressed the levels of histone lactylation in PA zygotes derived from vitrified mouse MII oocytes, but also promote it (H3K9la).

To research how TW-7 affected the histone lactylation levels of PA zygotes, we next examined the protein expression levels of EP300 and HDAC3 [27, 56] in PA zygotes of the fresh, vitrification, and V + TW-7 groups. Immunofluorescence displayed that the fluorescence intensity of EP300 in zygotes of the vitrification group was markedly reduced compared with those of the fresh group (*P* < 0.05), which was significantly elevated after TW-7 addition (*P* < 0.05) (Fig. 4C and D). The levels of HDAC3 fluorescence intensity had no difference in the three groups (*P* > 0.05) (Fig. 4C and D). Because lactate is the precursor for stimulating histone lactylation, we attempted to detect intracellular lactate levels in PA zygotes, but methods were not available. Given that lactate dehydrogenase LDHA and LDHB are the key enzymes to control lactate production, we then analyzed their levels of PA zygotes in the above three groups. Immunofluorescence displayed a significant decrease in LDHA and LDHB levels of vitrified zygotes compared to the fresh group (*P* < 0.05), and the TW-7 addition could recover their expression levels comparable to the fresh group (Fig. 4E and F). Similar results were observed in the expression of LDHB by WB (Fig. 4G and H). These results implied that TW-7 could rescue the decreased levels of histone lactylation in PA zygotes derived from vitrified mouse MII oocytes through affecting histone lactylation-related regulatory proteins.

### TW-7 rescued the suppressed G_1_/S transition and ZGA initiation of PA zygotes derived from vitrified MII oocytes potentially through up-regulation of histone lactylation

To clarify whether TW-7 rescued the suppressed G_1_/S transition and ZGA initiation of PA zygotes derived from vitrified MII oocytes through histone lactylation, firstly we conducted EdU and EU staining respectively to examine G_1_/S transition and new transcripts generation in PA zygotes of the vitrification, V + TW-7, V + TW-7 + oxamate, and V + lactate groups. Consistent with the above results, the TW-7 supplement in the vitrification group could markedly increase the proportion of the S phase zygotes and the proportion of new transcripts generation zygotes. Adding lactate (5 mmol/L) to the vitrification group achieved the same effect However, when supplemented the vitrification group with TW-7 and oxamate (5 mmol/L), LDH inhibitor, at the same time, the G_1_/S transition and new transcripts generation of PA zygotes were remarkably suppressed (Fig. S1B, 5A and B). These results indicated that TW-7 rescued the suppressed G_1_/S transition and new transcripts generation of PA zygotes derived from vitrified MII oocytes by regulating lactate levels.

**Fig. 5:**
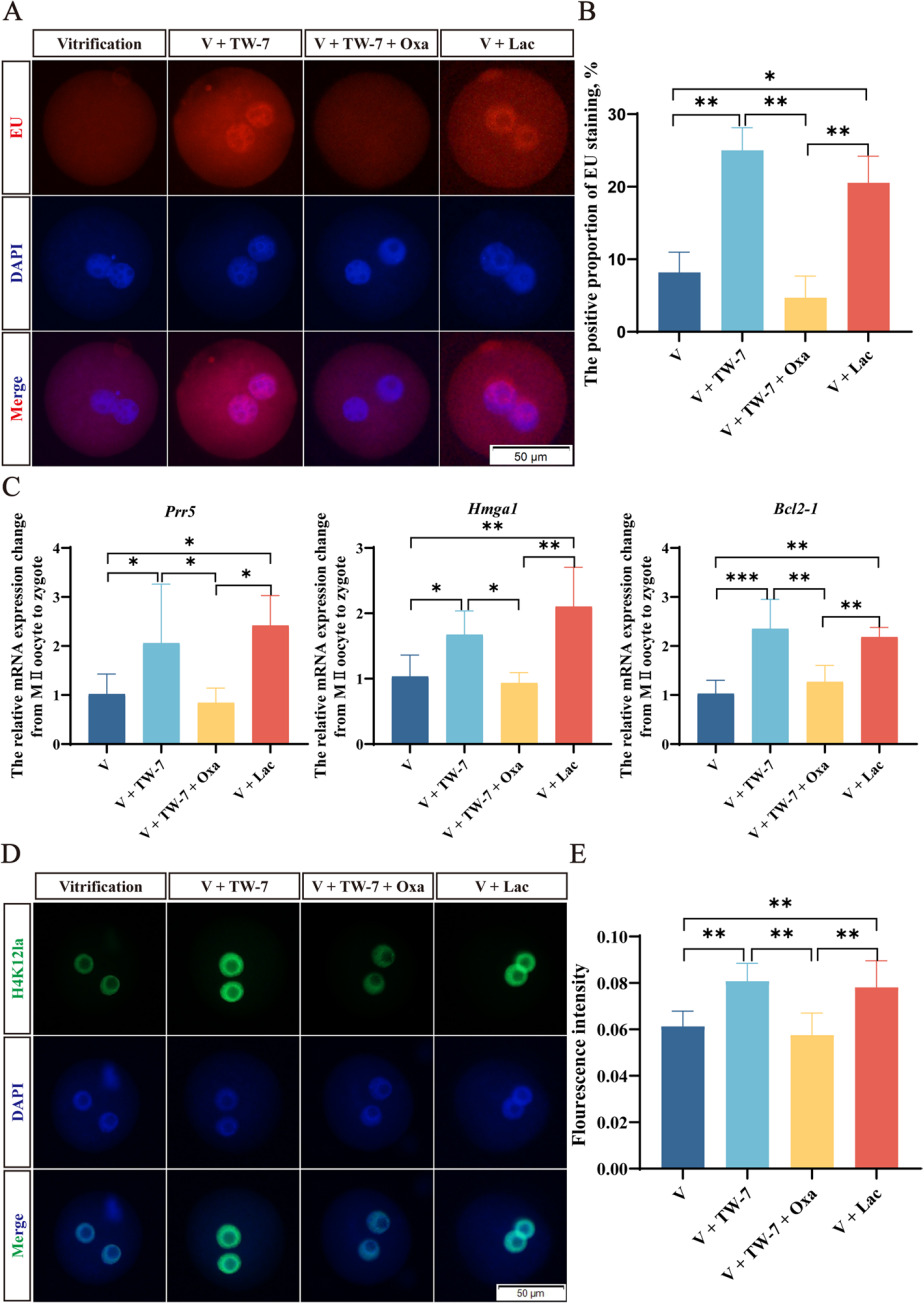
TW-7 may promote minor ZGA initiation in PA zygotes from vitrified mouse MII oocytes through histone lactylation. **A** Representative images of EU staining in PA zygotes of Vitrification, V + TW-7, V + TW-7 + Oxamate, and V + Lactate groups. Scale bar, 50 μm. **B** The positive proportion of EU staining in PA zygotes of Vitrification, V + TW-7, V + TW-7 + Oxamate, and V + Lactate groups. Vitrification (*n* = 36), V + TW-7 (*n* = 45), V + TW-7 + Oxamate (*n* = 48), V + Lactate (*n* = 40). **C** The relative mRNA expression changes from MII oocytes (Fresh group) to zygotes (Vitrification, V + TW-7, V + TW-7 + Oxamate, and V + Lactate groups). **D** Representative images of H4K12la protein expression in PA zygotes of Vitrification, V + TW-7, V + TW-7 + Oxamate, and V + Lactate groups. Scale bar, 50 μm. **E** The fluorescence intensity of H4K12la in PA zygotes of Vitrification, V + TW-7, V + TW-7 + Oxamate, and V + Lactate groups. Number of PA zygotes involved in Pan Kla immunofluorescence staining: Vitrification (*n* = 38), V + TW-7 (*n* = 34), V + TW-7 + Oxamate (*n* = 43), V + Lactate (*n* = 29). Data in (**B**), (**C**), and (**E**) were presented as mean percentage (mean ± SEM) of at least three independent experiments. ^*^P < 0.05, ^**^P < 0.01, ^***^P < 0.001

Next, the qRT-PCR was conducted to detect the mRNA expression levels of minor ZGA genes *Prr5*, *Hmga1,* and *Bcl2-1* in different treatment groups. Given that the general transcript level of MII oocyte was not affected by cryopreservation [55], we used the mRNA expression of the above minor ZGA genes in fresh MII oocytes to evaluate whether they were activated in different treatment groups. The results showed that the relative mRNA expression of *Prr5*, *Hmga1,* and *Bcl2-1* in the vitrification group was equal to fresh MII oocytes, and TW-7 or lactate (5 mmol/L) addition could increase the minor ZGA gene expression, which indicated that minor ZGA was suppressed in PA zygotes from vitrified MII oocytes but TW-7 or lactate could rescue it (Fig. 5C). When supplemented the vitrification group with TW-7 and oxamate (5 mmol/L) at the same time, the relative mRNA expression of *Prr5*, *Hmga1,* and *Bcl2-1* was reduced to the same level as vitrification group (*P* > 0.05) (Fig. 5C). The above results implied that TW-7 could rescue ZGA initiation of PA zygotes derived from vitrified MII oocytes by regulating lactate level.

Subsequently, we examined the levels of H4K12la in the above four groups. The immunofluorescence intensity of H4K12la in PA zygotes was significantly higher in the V + TW-7 or V + lactate groups than in the vitrification or V + TW-7 + oxamate groups (*P* < 0.01) (Fig. 5D and E), which revealed that TW-7 rescued the suppressed G_1_/S transition and ZGA initiation of PA zygotes derived from vitrified MII oocytes probably through up-regulation of histone lactylation.

### TW-7 promoted PA embryo development derived from vitrified MII oocytes potentially by regulating histone lactylation

**Fig. 6:**
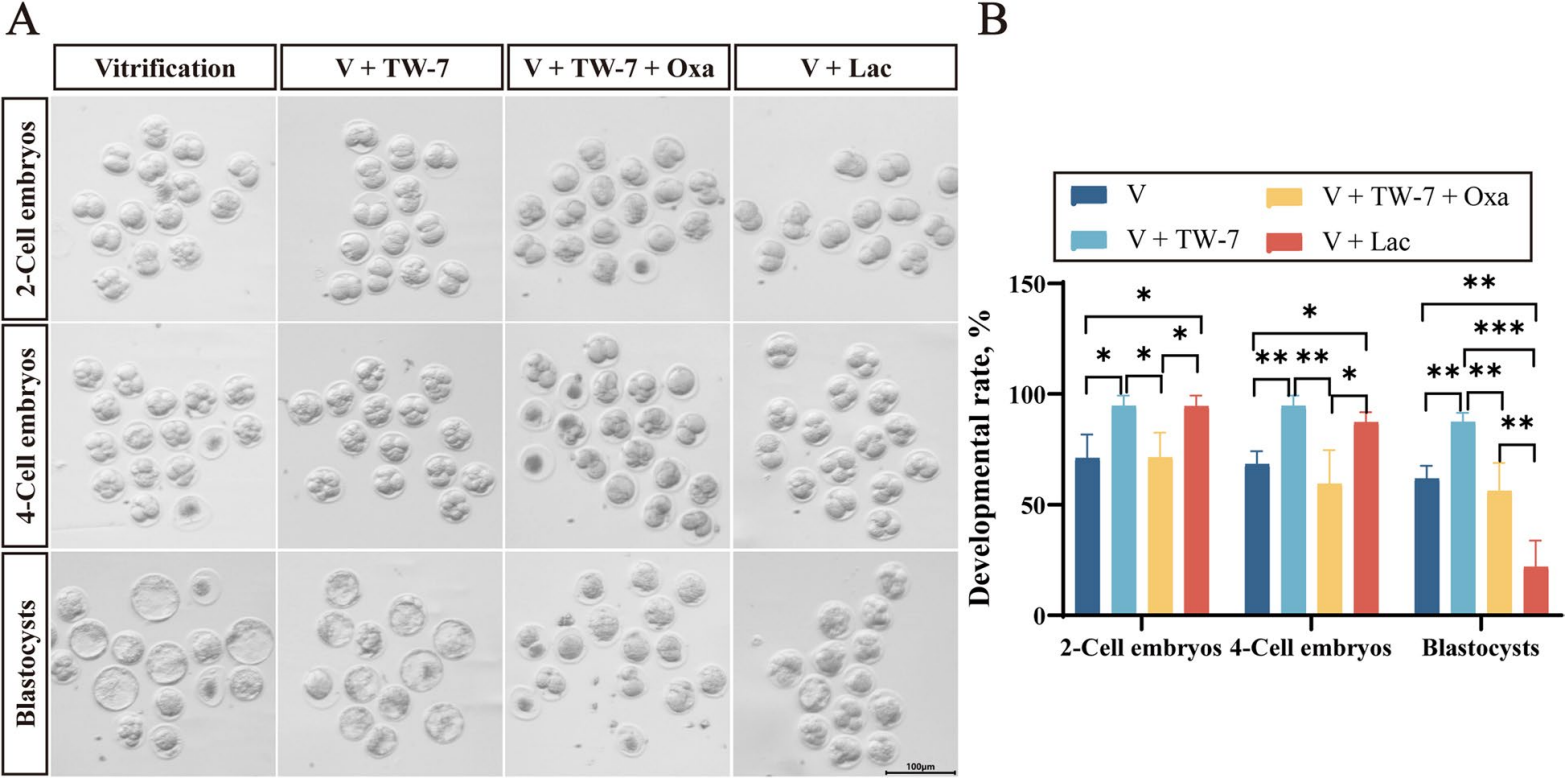
TW-7 promoted PA embryo development derived from vitrified MII oocytes potentially by regulating histone lactylation. **A** Representative images of 2-cell embryos, 4-cell embryos, and blastocysts in the Vitrification, V + TW-7, V + TW-7 + Oxamate, and V + Lactate groups. Scale bar, 100 μm. **B** The percentage of embryo development rate of oocytes in Vitrification, V + TW-7, V + TW-7 + Oxamate, and V + Lactate groups. Vitrification (*n* = 42), V + TW-7 (*n* = 44), V + TW-7 + Oxamate (*n* = 44), V + Lactate (*n* = 44). Data in (**B**) were presented as mean percentage (mean ± SEM) of at least three independent experiments. ^*^P < 0.05, ^**^P < 0.01, ^***^P < 0.001

To investigate whether TW-7 could promote preimplantation embryo development of the PA zygotes derived from vitrified MII oocytes by regulating histone lactylation, we examined the embryo development rates in the vitrification, V + TW-7, V + TW-7 + oxamate, and V + lactate groups. As shown in Fig. 6C and D, the suppressed preimplantation embryo development in the vitrification group was markedly attenuated by TW-7 supplementation (*P* < 0.05), consistent with the findings mentioned above. The addition of 5 mmol/L oxamate to the V + TW-7 group resulted in a significant decrease in the 2-cell embryo, 4-cell embryo, and blastocyst rates (*P* < 0.05), and was not significantly (*P* > 0.05) different from the vitrification group. Besides, the percentage of vitrified MII oocytes developing to 2-cell and 4-cell embryos was significantly higher in the V + lactate (5 mmol/L) group than in the vitrification group (*P* < 0.05), and was not significantly different from the V + TW-7 group (*P* > 0.05). Unexpectedly, the blastocyst rate of the V + lactate group was extremely lower than other three groups (*P* < 0.01) (Fig. 6A and B). These results indicated that TW-7 promoted the preimplantation embryo development of PA zygotes from vitrified MII oocytes by regulating histone lactylation.

### TW-7 improved the level of histone lactylation of the PA zygotes derived from vitrified mouse MII oocytes mainly through antioxidant pathway

**Fig. 7:**
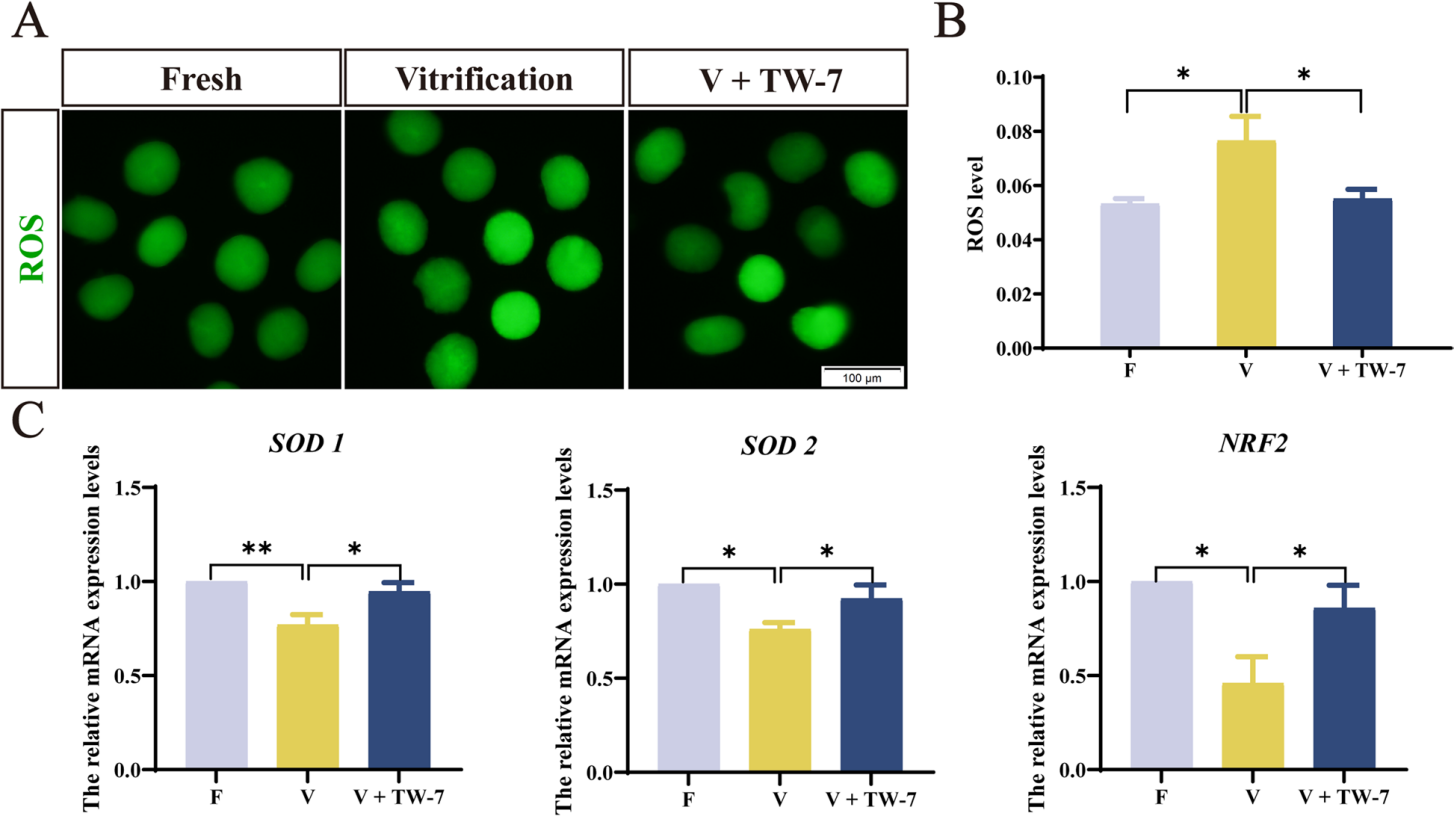
TW-7 modulates antioxidant levels in PA embryos from vitrified mouse MII oocytes. **A** Representative images of zygotes with DCHFDA staining. Scale bar, 100 μm. **B** The fluorescence intensity of ROS levels in zygotes. Fresh (*n* = 33), Vitrification (*n* = 43), V + TW-7 (*n* = 43). **C** The relative mRNA expression levels of antioxidant genes *SOD1*, *SOD2*, and *NRF2* of zygotes and 2-cell embryos in the Fresh, vitrification, and V + TW-7 groups. Data in (**B**) and (**C**) were presented as mean percentage (mean ± SEM) of at least three independent experiments. ^*^P < 0.05, ^**^P < 0.01

To explore whether TW-7 elevated histone lactylation levels of PA zygotes derived from vitrified mouse MII oocytes through the antioxidant pathway, we firstly examined the ROS levels of PA zygotes in the fresh, vitrification, and V + TW-7 groups. As shown in Fig. 7A and B, the ROS levels of PA zygotes (9 hpa) were significantly higher in the vitrification group than in the fresh group (*P* < 0.05). When TW-7 was added to the vitrification group, the fluorescence intensity of ROS was significantly decreased (*P* < 0.05), comparably to the fresh group (*P* > 0.05). These results displayed that TW-7 reduced the level of ROS in PA zygotes derived from vitrified MII oocytes.

Next, we examined the mRNA expression level of antioxidant genes such as *SOD1*, *SOD2,* and *Nrf2* in PA zygotes. As shown in Fig. 7C, the afore-said genes all showed significantly reduced mRNA expression levels in the vitrification group than in the fresh group (*P* < 0.05). The mRNA expression levels of *SOD1*, *SOD2,* and *Nrf2* elevated markedly when TW-7 was supplemented to the vitrification group (*P* < 0.05), which was indifferent to the fresh groups (*P* > 0.05) (Fig. 7C). These results suggested that TW-7 alleviated oxidative stress in PA zygotes induced by vitrification.

**Fig. 8:**
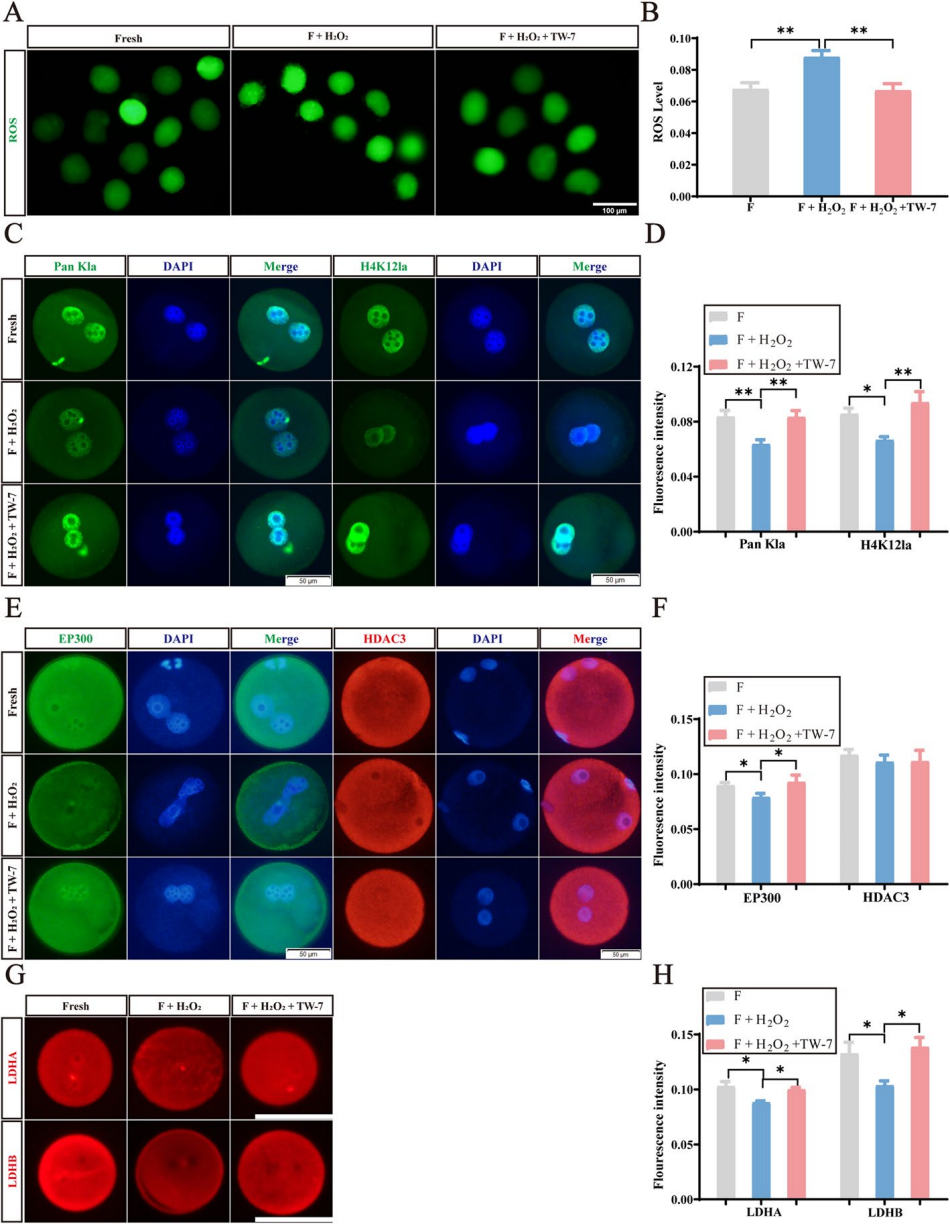
TW-7 may increase the level of histone lactylation of the PA zygotes derived from vitrified mouse MII oocytes via antioxidant pathways. **A** Representative images of DCHFDA staining in PA zygotes of Fresh, F + H_2_O_2_ and F + H_2_O_2_ + TW-7 groups. Scale bar, 50 μm. **B** The fluorescence intensity of ROS levels in PA zygotes of Fresh, F + H_2_O_2_, F + H_2_O_2_ + TW-7 groups. Fresh (*n* = 48), F + H_2_O_2_ (*n* = 47), F + H_2_O_2_ + TW-7 (*n* = 44). **C** Representative images of Pan Kla and H4K12la protein expression in PA zygotes of Fresh, F + H_2_O_2_, F + H_2_O_2_ + TW-7 groups. Scale bar, 50 μm. **D** The fluorescence intensity of LDHA and LDHB in PA zygotes of Fresh, F + H_2_O_2_, F + H_2_O_2_ + TW-7 groups. Number of PA zygotes involved in Pan Kla immunofluorescence staining: Fresh (*n* = 57), F + H_2_O_2_ (*n* = 54), F + H_2_O_2_ + TW-7 (*n* = 45). Number of PA zygotes involved in H4K12la immunofluorescence staining: Fresh (*n* = 39), F + H_2_O_2_ (*n* = 45), F + H_2_O_2_ + TW-7 (*n* = 40). **E** Representative images of EP300 and HDAC3 protein expression in PA zygotes of Fresh, F + H_2_O_2_, F + H_2_O_2_ + TW-7 groups. Scale bar, 50 μm. **F** The fluorescence intensity of EP300 and HDAC3 in PA zygotes of Fresh, F + H_2_O_2_, F + H_2_O_2_ + TW-7 groups. Number of PA zygotes involved in EP300 immunofluorescence staining: Fresh (*n* = 37), F + H_2_O_2_ (*n* = 31), F + H_2_O_2_ + TW-7 (*n* = 36). Number of PA zygotes involved in HDAC3 immunofluorescence staining: Fresh (*n* = 28), F + H_2_O_2_ (*n* = 33), F + H_2_O_2_ + TW-7 (*n* = 30). **G** Representative images of LDHA and LDHB protein expression in PA zygotes of Fresh, F + H_2_O_2_, F + H_2_O_2_ + TW-7 groups. Scale bar, 100 μm. **H** The fluorescence intensity of LDHA and LDHB in PA zygotes of Fresh, F + H_2_O_2_, F + H_2_O_2_ + TW-7 groups. Number of PA zygotes involved in LDHA immunofluorescence staining: Fresh (*n* = 36), F + H_2_O_2_ (*n* = 38), F + H_2_O_2_ + TW-7 (*n* = 38). Number of PA zygotes involved in LDHB immunofluorescence staining: Fresh (*n* = 51), F + H_2_O_2_ (*n* = 50), F + H_2_O_2_ + TW-7 (*n* = 52). Data in (**B**), (**D**), (**F**), and (**H**) were presented as mean percentage (mean ± SEM) of at least three independent experiments. ^*^P < 0.05, ^**^P < 0.01

In addition, we induced oxidative damage by H_2_O_2_ to imitate oxidative stress caused by vitrification. As shown in Fig. 8A and B, the ROS fluorescence intensity of the H_2_O_2_-exposed zygotes was significantly higher compared to the fresh group (*P* < 0.05). When TW-7 was added to the H_2_O_2_-treated group, the fluorescence intensity of ROS was significantly reduced (*P* < 0.01), displaying no difference from that of the fresh group (*P* > 0.05), manifesting that TW-7 also relieved oxidative stress inducing by H_2_O_2_. Furthermore, we found that the fluorescence intensity of Pan Kla, H4K12la, EP300, LDHA, and LDHB was significantly reduced in H_2_O_2_-exposed zygotes (*P* < 0.05), whereas the fluorescence intensity of the above proteins was significantly increased by the addition of TW-7 (*P* < 0.05), and was not significantly different from those of the fresh group (*P* > 0.05) (Fig. 8C–H). These findings revealed that TW-7 improved the level of histone lactylation of the PA zygotes derived from vitrified MII oocytes principally through the antioxidant pathway. However, the fluorescence intensity of HDAC3 in the fresh, H_2_O_2_-treated, and F + H_2_O_2_ + TW-7 groups had no difference (*P* > 0.05) (Fig. 8C and D), suggesting that oxidative stress may not inhibit histone lactylation modification via HDAC3.

In conclusion, TW-7 may increase histone lactylation-related regulatory proteins such as LDHA, LDHB, and EP300 in PA zygotes derived from vitrified MII oocytes via the antioxidant pathway, which in turn improved histone lactylation level.

## Discussion

Oocyte vitrification is increasingly used for female fertility preservation. However, as previously described, vitrification may cause damage to the endogenous antioxidant system and redox homeostasis imbalance in several mammalian oocytes [57–65], which puts a great adverse impact on the developmental potential of the oocyte [21, 58–60]. In the present study, we found that the PA zygotes derived from vitrified mouse MII oocytes showed the increased oxidative stress. In addition, vitrification also led to delayed progression of pronucleus formation, which was consistent with previous findings [12]. Previous researches suggested that exogenous antioxidants, such as melatonin [12, 17, 45] and resveratrol [20, 66, 67], could improve vitrified oocyte quality and developmental potential. In this study, similarly, we found that supplementation of TW-7 in oocyte warming, recovery, PA, and embryo culture medium could significantly alleviate oxidative damage caused by vitrification in oocytes and improve the development rate of PA embryos from vitrified MII oocytes. Given that TW-7 can be easily synthesized at relatively low cost [43, 44], it is cost-effective or easily obtainable compared to the above-mentioned antioxidants.

Current research also investigated the mechanism by which oxidative stress adversely affected the embryo development. Previous studies suggested that mitochondrial dysfunction induced by oocyte vitrification exacerbated ROS production [21, 68]. The excessive ROS in turn inhibited the activity of glycolytic pyruvate kinase M2 (PKM2) by promoting its phosphorylation, which consequently reduced the production of the glycolytic products like lactate [40, 41]. In consistence with the above reports, this study also found lactate dehydrogenase (LDHA, LDHB) protein levels were significantly reduced in zygotes derived from cryopreserved mouse MII oocytes, indicating that vitrification could inhibit the production of lactate in direct and indirect ways. Besides, previously published studies found that LDHA and LDHB knockdown in macrophages repressed lactate production, and the level of histone lactylation was consequently reduced [27], which implied the correlation between the level of lactate and histone lactylation. In this paper, we found that vitrification concurrently affected the protein levels of LDHA, LDHB, and histone lactylation modification-related enzyme EP300 in zygotes. The decreased levels of histone lactylation (Pankla and H4K12la) may result from decreased LDHA, LDHB, and EP300 of PA zygotes. Moreover, the oxidative model suggested that TW-7 may improve the level of histone lactylation in PA zygotes from vitrified mouse MII oocytes via the antioxidant pathway.

As a novel histone post-translational modification, histone lactylation has been identified to promote cell growth and development by regulating target gene transcription [69–71]. For example, down-regulating H4K12la inhibited CCNB1 transcription thereby retarding DNA replication and the cell cycle [34]. Addition of lactate activated the transcription of ZGA genes such as the *Zscan4* gene family by increasing the level of H3K18la, which contributed to the proliferation and development of mouse embryonic stem cells [35]. Lactate deprivation resulted in H3K18la loss, thus causing major ZGA failure and 2-cell embryo development arrest in mice and humans [36]. Besides, the vitrification of porcine zygotes significantly reduced the transcriptional activity of the ZGA genes in 4-cell and 8-cell embryos [72]. Therefore, we hypothesized that vitrification may down-regulate histone lactylation, and then repress ZGA gene transcription and consequently hamper the embryo development. In this study, we found that supplementing lactate or TW-7 in oocyte warming, recovery, PA, and embryo culture medium significantly promoted the expression level of H4K12la and ZGA genes in PA zygotes derived from vitrified mouse MII oocytes, as well as their preimplantation embryo development. However, the addition of oxamate (LDH inhibitor) and TW-7 at the same time resulted in the disappearance of the above beneficial effects, which revealed that TW-7 may alleviate the repressed development of PA embryos from vitrified mouse MII oocytes by up-regulating the expression level of H4K12la. However, the beneficial effect of lactate addition in embryo development was displayed in 2-cell embryos and 4-cell embryos, but not in blastocysts. One possible speculation is that the embryo metabolic patterns shift gradually from the pyruvate and lactate metabolism to oxidative phosphorylation after densification [73]. However, more accurate mechanisms require further study.

It is notable that the zygotes originated from PA rather than IVF/ICSI in this paper. Therefore, it needs to be further verified whether TW-7 can improve the developmental potential of mouse IVF/ICSI embryos derived from vitrified MII oocytes. Besides, considering species difference, whether TW-7 can rescue the impaired embryonic development in other mammals caused by egg freezing and its mechanism remains to be studied.

## Conclusion

**Fig. 9:**
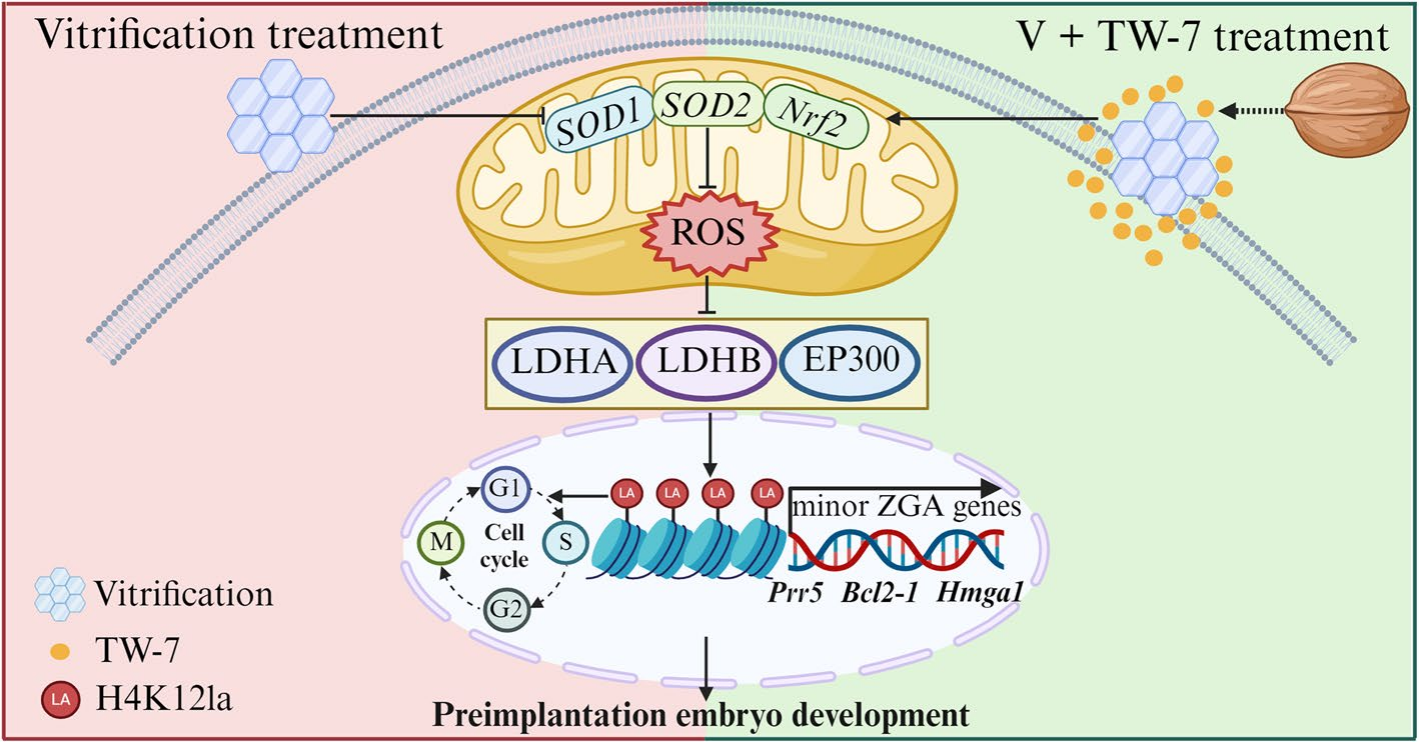
The walnut-derived peptide TW-7 improves mouse parthenogenetic embryo development of vitrified MII oocytes potentially by promoting histone lactylation. Created with BioRender.com

Our researches revealed that the cryopreservation of mouse MII oocytes could induce an increase in oxidative stress, and consequently a decrease in the expression levels of histone lactylation modification-related enzyme (LDHA, LDHB, EP300) and the repressed histone lactylation in PA zygotes, which potentially affected the minor ZGA initiation, leading to the developmental delay of PA embryos at the early cleavage stage and subsequent embryo development arrest. TW-7 markedly improved the preimplantation embryo development of PA embryos from vitrified MII oocytes through alleviating oxidative stress-induced histone lactylation repression. (Fig. 9).

### Supplementary Information

**Additional file 1:** **Fig. S1** TW-7 may promote the G1/S transition in PA zygotes from vitrified mouse MII oocytes through histone lactylation. **A** The percentage of entering the S phase of PA zygotes (9 hpa) in the Fresh, Vitrification, and V + TW-7 groups. Fresh (*n* = 39), Vitrification (*n* = 39), V + TW-7 (*n* = 49). **B** The percentage of entering the S phase of PA zygotes (9 hpa) in the Vitrification, V + TW-7, V + TW-7 + Oxamate, and V + Lactate groups. Vitrification (*n* = 41), V + TW-7 (*n* = 44), V + TW-7 + Oxamate (*n* = 43), V + Lactate (n = 44). Data in (**A**) and (**B**) were presented as mean percentage (mean ± SEM) of at least three independent experiments. ^*^P < 0.05, ^**^P < 0.01, ^***^P < 0.001. **Fig. S2** TW-7 regulates the protein expression levels of histone lactylation modification site in PA zygotes derived from vitrified mouse MII oocytes. **A** Representative images of H3K18la and H3K9la protein expression in PA zygotes of Fresh, Vitrification, and V + TW-7 groups. Scale bar, 50 μm. **B** The fluorescence intensity of H3K18la and H3K9la in PA zygotes of Fresh, Vitrification, and V + TW-7 groups. Number of PA zygotes involved in H3K18la immunofluorescence staining: Fresh (*n* = 57), Vitrification (*n* = 43), V + TW-7 (*n* = 59). Number of PA zygotes involved in H3K9la immunofluorescence staining: Fresh (*n* = 20), Vitrification (*n* = 19), V + TW-7 (*n* = 19). Data in (**B**) were presented as mean percentage (mean ± SEM) of at least three independent experiments. ^*^P < 0.05.

## Data Availability

Data will be made available on request.

## Abbreviations

AEWC: Animal ethical and welfare committee
DEGs: Differentially expressed genes
EdU: 5-Ethynyl-20-deoxyuridine
EU: 5-Ethynyl uridine
GO: Gene Ontology
hCG: Human chorionic gonadotropin
hpa: Hours post activation
HTF: Human tubal fluid
H2DCFDA: 2, 7-Dichlorodihydrofluorescein diacetate
IVC: In vitro culture
IVF: In vitro fertilization
KSOM-AA: Simple optimization of medium contained K ions and amino acid
LDHA: Lactate dehydrogenase A
LDHB: Lactate dehydrogenase B
MII: Metaphase II
MMP: Mitochondrial membrane potential
OPS: Open-pulled straws
PA: Parthenogenetic activation
PBS: Phosphate buffer saline
PMSG: Equine chorionic gonadotropin
qTR-PCR: Quantitative reverse transcription polymerase chain reaction
ROS: Reactive oxygen species
TW-7: The walnut-derived peptide Thr-Trp-Leu-Pro-Leu-Pro-Arg
ZGA: Zygotic genome activation
