# Supplementary figures and images for: The walnut-derived peptide TW-7 improves mouse parthenogenetic embryo development of vitrified MII oocytes potentially by promoting histone lactylation
Source: J Anim Sci Biotechnol. 2024 Jun 11;15:86. doi: 10.1186/s40104-024-01045-0 (PMC11165821; doi:10.1186/s40104-024-01045-0)

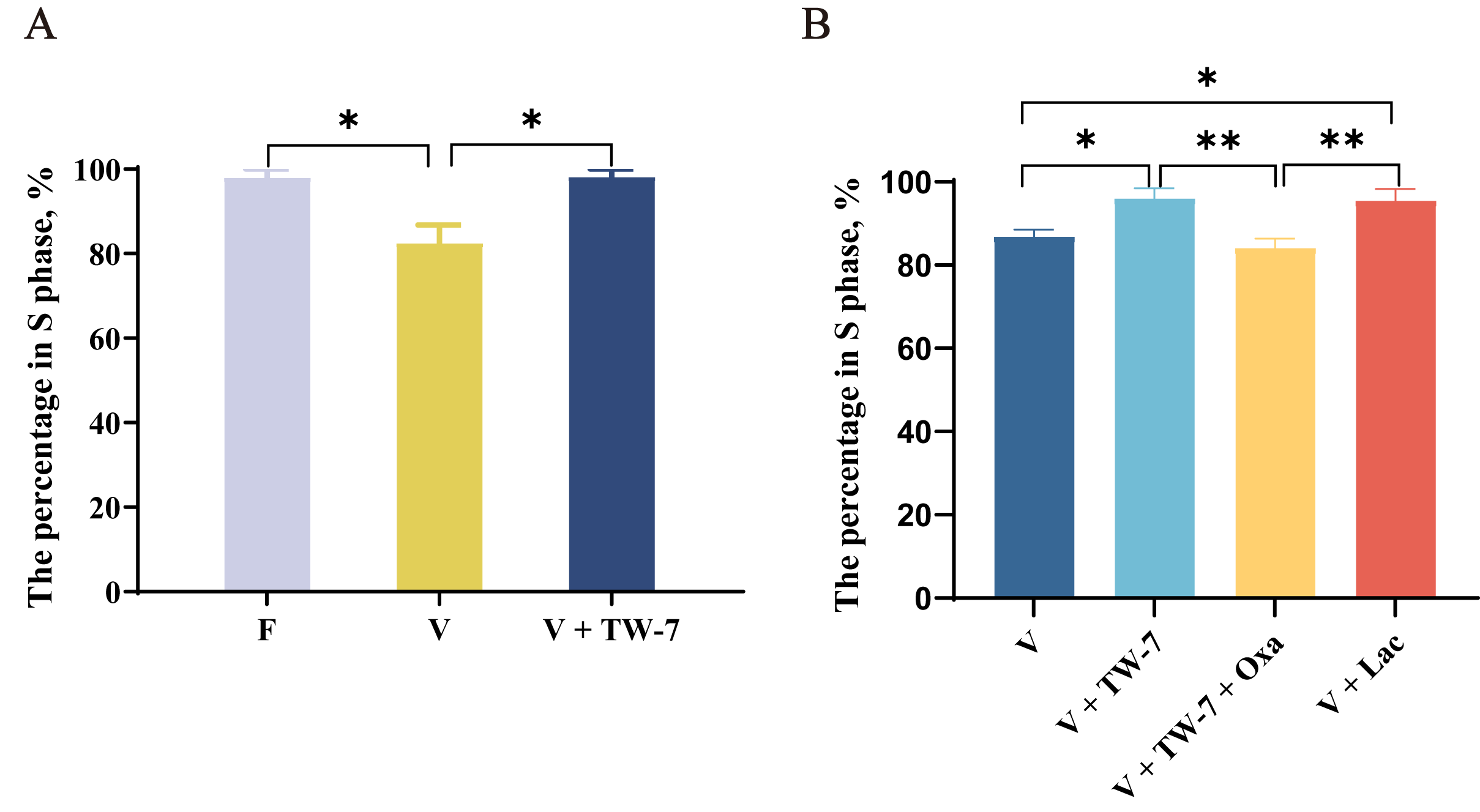

Supplement: Supplementary file 1 — Additional file 1: Fig. S1 TW-7 may promote the G1/S transition in PA zygotes from vitrified mouse MII oocytes through histone lactylation. A The percentage of entering the S phase of PA zygotes (9 hpa) in the Fresh, Vitrification, and V + TW-7 groups. Fresh (n = 39), Vitrification (n = 39), V + TW-7 (n = 49). B The percentage of entering the S phase of PA zygotes (9 hpa) in the Vitrification, V + TW-7, V + TW-7 + Oxamate, and V + Lactate groups. Vitrification (n = 41), V + TW-7 (n = 44), V + TW-7 + Oxamate (n = 43), V + Lactate (n = 44). Data in (A) and (B) were presented as mean percentage (mean ± SEM) of at least three independent experiments. *P < 0.05, **P < 0.01, ***P < 0.001. Fig. S2 TW-7 regulates the protein expression levels of histone lactylation modification site in PA zygotes derived from vitrified mouse MII oocytes. A Representative images of H3K18la and H3K9la protein expression in PA zygotes of Fresh, Vitrification, and V + TW-7 groups. Scale bar, 50 μm. B The fluorescence intensity of H3K18la and H3K9la in PA zygotes of Fresh, Vitrification, and V + TW-7 groups. Number of PA zygotes involved in H3K18la immunofluorescence staining: Fresh (n = 57), Vitrification (n = 43), V + TW-7 (n = 59). Number of PA zygotes involved in H3K9la immunofluorescence staining: Fresh (n = 20), Vitrification (n = 19), V + TW-7 (n = 19). Data in (B) were presented as mean percentage (mean ± SEM) of at least three independent experiments. *P < 0.05. [file 40104_2024_1045_MOESM1_ESM.zip › 40104_2024_1045_MOESM1_ESM.tif]

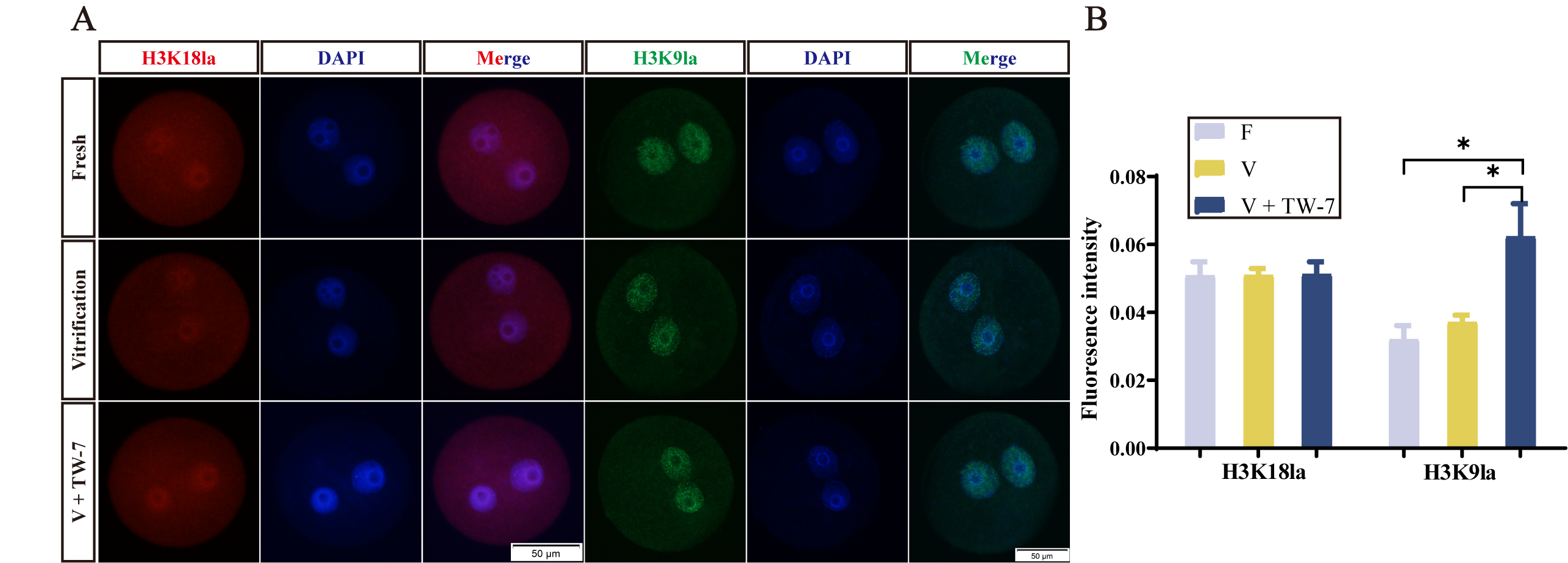

Supplement: Supplementary file 1 — Additional file 1: Fig. S1 TW-7 may promote the G1/S transition in PA zygotes from vitrified mouse MII oocytes through histone lactylation. A The percentage of entering the S phase of PA zygotes (9 hpa) in the Fresh, Vitrification, and V + TW-7 groups. Fresh (n = 39), Vitrification (n = 39), V + TW-7 (n = 49). B The percentage of entering the S phase of PA zygotes (9 hpa) in the Vitrification, V + TW-7, V + TW-7 + Oxamate, and V + Lactate groups. Vitrification (n = 41), V + TW-7 (n = 44), V + TW-7 + Oxamate (n = 43), V + Lactate (n = 44). Data in (A) and (B) were presented as mean percentage (mean ± SEM) of at least three independent experiments. *P < 0.05, **P < 0.01, ***P < 0.001. Fig. S2 TW-7 regulates the protein expression levels of histone lactylation modification site in PA zygotes derived from vitrified mouse MII oocytes. A Representative images of H3K18la and H3K9la protein expression in PA zygotes of Fresh, Vitrification, and V + TW-7 groups. Scale bar, 50 μm. B The fluorescence intensity of H3K18la and H3K9la in PA zygotes of Fresh, Vitrification, and V + TW-7 groups. Number of PA zygotes involved in H3K18la immunofluorescence staining: Fresh (n = 57), Vitrification (n = 43), V + TW-7 (n = 59). Number of PA zygotes involved in H3K9la immunofluorescence staining: Fresh (n = 20), Vitrification (n = 19), V + TW-7 (n = 19). Data in (B) were presented as mean percentage (mean ± SEM) of at least three independent experiments. *P < 0.05. [file 40104_2024_1045_MOESM1_ESM.zip › 40104_2024_1045_MOESM2_ESM.tif]
