# Supplementary material for: The walnut-derived peptide TW-7 improves mouse parthenogenetic embryo development of vitrified MII oocytes potentially by promoting histone lactylation
Source: J Anim Sci Biotechnol. 2024 Jun 11;15:86. doi: 10.1186/s40104-024-01045-0 (PMC11165821; doi:10.1186/s40104-024-01045-0)
Supplement: Supplementary file 1 — Additional file 1: Fig. S1 TW-7 may promote the G1/S transition in PA zygotes from vitrified mouse MII oocytes through histone lactylation. A The percentage of entering the S phase of PA zygotes (9 hpa) in the Fresh, Vitrification, and V + TW-7 groups. Fresh (n = 39), Vitrification (n = 39), V + TW-7 (n = 49). B The percentage of entering the S phase of PA zygotes (9 hpa) in the Vitrification, V + TW-7, V + TW-7 + Oxamate, and V + Lactate groups. Vitrification (n = 41), V + TW-7 (n = 44), V + TW-7 + Oxamate (n = 43), V + Lactate (n = 44). Data in (A) and (B) were presented as mean percentage (mean ± SEM) of at least three independent experiments. *P < 0.05, **P < 0.01, ***P < 0.001. Fig. S2 TW-7 regulates the protein expression levels of histone lactylation modification site in PA zygotes derived from vitrified mouse MII oocytes. A Representative images of H3K18la and H3K9la protein expression in PA zygotes of Fresh, Vitrification, and V + TW-7 groups. Scale bar, 50 μm. B The fluorescence intensity of H3K18la and H3K9la in PA zygotes of Fresh, Vitrification, and V + TW-7 groups. Number of PA zygotes involved in H3K18la immunofluorescence staining: Fresh (n = 57), Vitrification (n = 43), V + TW-7 (n = 59). Number of PA zygotes involved in H3K9la immunofluorescence staining: Fresh (n = 20), Vitrification (n = 19), V + TW-7 (n = 19). Data in (B) were presented as mean percentage (mean ± SEM) of at least three independent experiments. *P < 0.05. [file 40104_2024_1045_MOESM1_ESM.zip › Supplementary figure legends_ESM.doc]

**Supplemental Fig. S1** TW-7 may promote the G_1_/S transition in PA zygotes from vitrified mouse MII oocytes through histone lactylation. (A) The percentage of entering the S phase of PA zygotes (9 hpa) in the Fresh, Vitrification, and V + TW-7 groups. Fresh (n = 39), Vitrification (n = 39), V + TW-7 (n = 49). (B) The percentage of entering the S phase of PA zygotes (9 hpa) in the Vitrification, V + TW-7, V + TW-7 + Oxamate, and V + Lactate groups. Vitrification (n = 41), V + TW-7 (n = 44), V + TW-7 + Oxamate (n = 43), V + Lactate (n = 44). Data in (A) and (B) were presented as mean percentage (mean ± SEM) of at least three independent experiments. **P* < 0.05, ***P* < 0.01, ****P* < 0.001.

**Supplemental Fig. S2** TW-7 regulates the protein expression levels of histone lactylation modification site in PA zygotes derived from vitrified mouse MII oocytes. (A) Representative images of H3K18la and H3K9la protein expression in PA zygotes of Fresh, Vitrification, and V + TW-7 groups. Scale bar, 50 μm. (B) The fluorescence intensity of H3K18la and H3K9la in PA zygotes of Fresh, Vitrification, and V + TW-7 groups. Number of PA zygotes involved in H3K18la immunofluorescence staining: Fresh (n = 57), Vitrification (n = 43), V + TW-7 (n = 59). Number of PA zygotes involved in H3K9la immunofluorescence staining: Fresh (n = 20), Vitrification (n = 19), V + TW-7 (n = 19). Data in (B) were presented as mean percentage (mean ± SEM) of at least three independent experiments. **P* < 0.05.
